# Great apes selectively retrieve relevant memories to guide action

**DOI:** 10.1038/s41598-020-69607-6

**Published:** 2020-07-28

**Authors:** Katarzyna Bobrowicz, Mikael Johansson, Mathias Osvath

**Affiliations:** 1 Department of Philosophy and Cognitive Science, Lund University, Lund, Sweden; 2 Department of Psychology, Lund University, Lund, Sweden

**Keywords:** Psychology, Animal behaviour

## Abstract

Memory allows us to draw on past experiences to inform behaviour in the present. However, memories rarely match the situation at hand exactly, and new situations regularly trigger multiple related memories where only some are relevant to act upon. The flexibility of human memory systems is largely attributed to the ability to disregard irrelevant, but salient, memories in favour of relevant ones. This is considered an expression of an executive function responsible for suppressing irrelevant memories, associated with the prefrontal cortex. It is unclear to what extent animals have access to this ability. Here, we demonstrate, in a series of tool-use tasks designed to evoke conflicting memories, that chimpanzees and an orangutan suffer from this conflict but overcome it in favour of a more relevant memory. Such mnemonic flexibility is among the most advanced expressions of executive function shown in animals to date and might explain several behaviours related to tool-use, innovation, planning and more.

## Introduction

Memory systems have evolved to enable us to draw on past experiences to understand and solve problems at hand. When we encounter an unprecedented situation, we rely on memories that overlap in perceptual or functional features with the current situation^1,2^, and we have to extract the relevant features to solve the problem. Such extraction of relevant elements of past experiences, rather than relying on perfect matches, is arguably one of the most adaptive functions of long-term memory, both declarative and nondeclarative^3,4^.

In humans, filtering out relevant features often comes at a cost. Memories of previous experiences are retrieved when certain cues in the environment overlap with existing memory traces^5,6^. Often though, a cue activates several memory traces, each of which matches some aspect of the retrieval cue, for example, a specific arrangement of shapes, colours, smells or causal relationships. This multiple activation leads to retrieval competition, as not all of these memory traces are relevant for the current situation. Such retrieval competition must be resolved, so that the goal-relevant target memory trace is used for an appropriate response. Overcoming irrelevant, but similar and salient, memory traces, is facilitated by the prefrontal cortex, and belongs to the cognitive executive function repertoire^7,8^. It has been shown that several animal taxa, from insects to birds and mammals, are prone to various forms of memory interference, in which memories interact and influence each other^9–11^. However, it is unclear whether any animals other than humans have executive functions of resolving conflicts of retrieval competition. In humans, such executive functions allow the maintenance and selective processing of concurrent mental representations which is critical to flexible cognition.

Animal memories have often been considered less flexible than those of humans. Mainly because it is argued that animals do not understand that their memories are *of* or *about* something, in the sense that they cannot reflect on their own memories as being memories; that is, they fail to reflect on their own mental states^12–15^. Memories can, in principle, guide behaviour without the need to reflect on these memories. In such cases, a retrieved memory becomes an immediate internal state that triggers a certain action, without an accompanying understanding that this state is in fact a representation of a past situation. Over-reliance on the strongest and most accessible memory trace has the potential to impede behavioural flexibility because it might be irrelevant. One way to test whether an animal is capable of representing relationships between memories and reality, is to discern whether the animal recognizes that its memories can be misrepresentations^12^. The most common empirical approach to meta-cognition in animals involves meta-memory tests aimed at establishing whether the animal can monitor its own memories and decide if memories are certain or uncertain. This ability has been shown in several paradigms in some species, such as great apes and macaques^16,17^. It is, however, difficult to discern whether this meta-cognitive ability results from an understanding that the memory is a misrepresentation, or if the animal just monitors its feeling of uncertainty, which is indeed what humans often do^13^. But conflict resolution between competing memory traces requires a different response option than that provided by meta-memory tests. In the latter, the animal can often opt out if it is uncertain of its memory (the opt -out alternative gives a smaller reward than the correct memory response, but an incorrect response leads to no reward). In a memory conflict resolution test, which we present here, the animal has to show not only that it recognizes that the initial salient memory trace is irrelevant for the task but also needs to actively overcome it in favour of another memory that leads to the correct solution. Whether this would be a better test of meta-memory is a matter for a theoretical debate, but it reveals a flexibility that cannot be revealed in previous paradigms. It is one thing to realize that your currently retrieved memory is inapplicable, and another to selectively identify a more relevant one.

To date, the cognitive processes responsible for technical innovations in animals are poorly understood, but it has been suggested that behavioural flexibility is essential^18^. We suggest that the type of memory conflict resolution tested in this study may be a key skill for such behavioural flexibility, as it allows for drawing on, combining and sorting between past experiences that differ in their usefulness to solve a novel problem.

In this study, we tested the ability to overcome competing, irrelevant memory traces in favour of relevant ones in five chimpanzees (*Pan troglodytes*) and one orangutan (*Pongo abelii*). Great apes are good candidates for the current study because, as mentioned above, they already show flexible memory retrieval, but they also show well-developed executive functions in many tasks, such as self-control^19^, reversal learning^20^, meta-memory^16^ and motor inhibition^21^. In several areas, they have long-term memory systems similar to humans, from temporal spans to the function of cueing and forgetting^22^. Importantly, great apes are known for their high levels of innovation both in captivity and in the wild^18,23–27^.

Whether great apes behave flexibly in the face of novel problems is under debate^28^. While some studies demonstrated that great apes can do so^26,29,30^, other showed that apes suffer conservative bias and functional fixedness^23–25,27^,that is, they keep to previous solutions when encountering novel problems. Perhaps, in such encounters, great apes rely on the most recent and therefore stronger memory traces^31–34^, even if they are no longer applicable because, contrary to humans, apes may not be able to overcome stronger memory traces in favour of weaker ones. Strong memories are more readily activated than weaker ones when presented with a matching retrieval cue. The accessibility and salience of a memory trace is driven by several factors, such as the recency and depth of encoding, and the overlap between the characteristics of the memory trace and the current task. Here, we examined how apes handle mnemonic conflict by increasing the salience of irrelevant, competing memories.

Previous studies have revealed that great apes can prioritize relevant aspects of a past experience over irrelevant ones^26,29^ and that they will use a past foraging strategy instead of more recently used one if the problem calls for it^25^. It has also been shown that other non-human primates will prioritize a more relevant touchscreen response over an irrelevant one, if they receive a visual cue *(colour or shape)*^35^*.* However, it is unclear whether any animals can prioritize one past experience (rather than features of the same experience) over another, when the tasks’ perceptual features cannot inform the functional relevance. In this case, one can suffer a conflict between two cued memories, in which the perceptual overlap is not congruent with the functional overlap; in other words, a conflict between a similar-looking but functionally irrelevant experience and a different-looking but functionally relevant experience. Overcoming such a conflict would be highly indicative of exercising an executive function that resolves retrieval competition.

Based on aforementioned studies on various executive functions and behavioural flexibility in apes we predicted that they, like humans, will suffer a conflict between two memories cued by a present problem and resolve the conflict in favour of a relevant memory. This seems to partly contradict previous findings on conservative bias and functional fixedness, however we believed that causally clear tasks will not result in such biases or strategies^26,29^. We used a so-called transfer test paradigm with a misleading irrelevant task learned between the two functionally overlapping tasks to ensure that the apes encoded conflicting memories. A transfer paradigm tests whether something which is experienced in one condition will be generalized to another condition that deviates to a certain extent from the first one. Often, the conditions are overlapping functionally, but not perceptually. The paradigm has frequently been used to test physical understanding and tool-using abilities, primarily in primates and corvids^36–40^. Importantly for the current study, it has been shown that chimpanzees can retain tool using skills learned several years ago and transfer these skills to a context that deviates from the learning context^40^.

Our basic set-up consisted of a sequence of three extractive foraging tasks, in which the last task tested resolution of memory competition; that is, whether the apes would be able to sort out the relevant memory traces from the irrelevant ones acquired in two previous extractive foraging tasks. The first task consisted of an apparatus that overlapped functionally, but not perceptually with the test task. The second task overlapped perceptually, but not functionally with the test task. Therefore, in the test, the apes had to overcome conflict from the strong perceptual overlap with the second task and retrieve the relevant target memories of the function acquired in the first one. As chimpanzees have previously been shown to understand the functional requirements of various apparatuses^41^, we expected that they would be able to identify the functional overlaps between the current extractive foraging tasks. To emulate real-life problems, we used apparatuses that required several motor patterns, and which contained several potential memory cues.

To increase interference due to retrieval competition, we ensured that the perceptually overlapping memory trace—the competitor—was more recently encoded than the functionally overlapping target memory. We also compared the subjects’ performance with a situation in which they had no conflicting memories, either by having only a functionally relevant experience or no experiences at all. We expected that the subjects’ performance would be differentially influenced by these three situations; namely, having no experiences at all would not allow for solving the test, having a functionally relevant experience would promote solving the test, and having two conflicting memories would hinder the success.

## Methods

### Overview

A counterbalanced within-subject design was used, and each subject was randomly assigned to a set of tasks and conditions. The control condition was never administered first, but otherwise the order of conditions was individualized and pseudorandomized. No criteria for data inclusion/exclusion were set, and there was no definition and policy for outliers. All data was included in the study and outliers were not detected. Each test trial, consisting of up to 10 attempts, was performed once. All testing was carried out by one experimenter who was known to the subjects.

To set up a competition between two memories, we introduced a test task that partially overlapped with each of two training tasks. The test task overlapped functionally with one training task (henceforth: *the functionally overlapping task*, the FOT), as they required the same tool and a similar motor pattern for solution (for details see “Apparatus”). The test task overlapped perceptually with the other training task (henceforth: *the perceptually overlapping task*, the POT). Each task involved opening a puzzle box with appropriate tools and retrieving food rewards from behind a transparent door. Whereas the FOT and the test could be opened with the same tool (henceforth: *the right tool*), the POT could also be opened with another tool, which, however, did not allow for solving the FOT and the test (henceforth: *the wrong tool*). The right and the wrong tool were sticks identical in material and dimensions, but only the right tool had two functional tips that allowed for opening the test task. Simply put, only the right tool was relevant for the solution of the problematic situation embodied by the test. The right and wrong tools were accompanied by a third tool that served as a distractor. This *useless* tool was a thin twig or a string with an appropriate length but not rigid enough to be useful^42,43^. The wrong tool was not available in the training on the FOT two reasons: (1) because of the subjects’ tendency toward tool destruction (a larger number of available tools gave an opportunity to destroy a larger number of tools and trade more tool pieces for food); (2) to maximize the perceptual overlap between the training on the POT and the test, as these problems were always accompanied by all three tools: the right, the wrong and the useless one.

Solving the tasks required using the right tool on those components of the puzzle box that were relevant for the solution; that is, that were necessary to interact with in order to open the box. Other components were defined as irrelevant for the solution. To measure the subject’s behaviour in the test, we quantified interactions between the right/wrong tool and the relevant/irrelevant components. In particular, we were interested in the interactions between the right tool and the relevant components (henceforth: *the relevant interactions)* because such interactions would indicate attending to the relevant aspects of the solution and the problem, respectively. Such attending, in turn, would indicate whether and to what extent the apes used the overlapping past situations to solve the problem at hand. As attending to the irrelevant aspects of the solution and the problem would indicate that the apes did not benefit from the past situations, we were also interested in the interactions between the wrong tool and the irrelevant components (henceforth: *the irrelevant interactions).*

**Figure 1:**
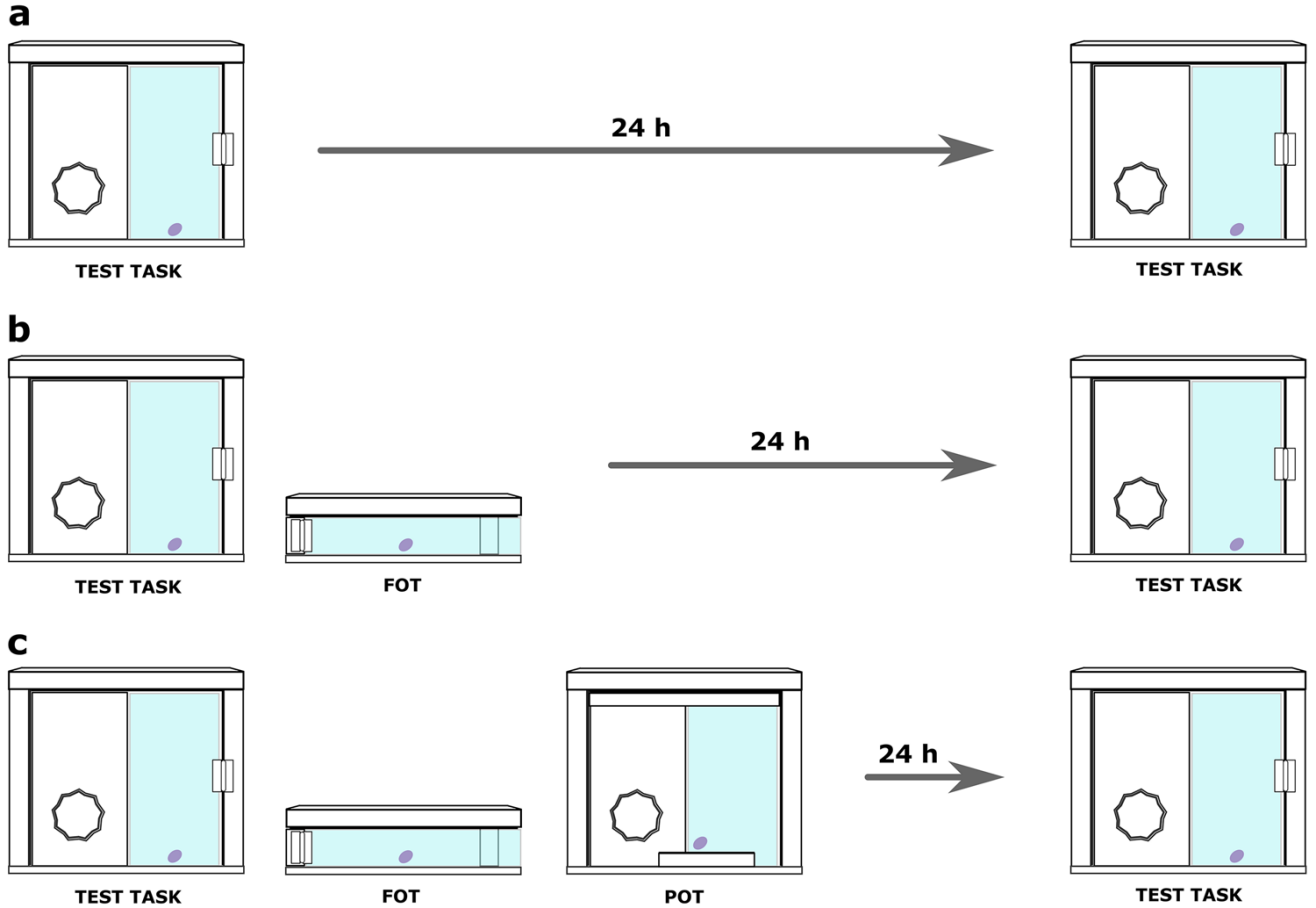
A display of the three conditions completed by each subject. (**a**) Control: the subject was exposed twice to the test task. A 24-h delay, but no trainings were introduced between the two exposures. (**b**) No-conflict: after the first exposure to the test task, the subject received a training on a functionally overlapping task (FOT). After it reached the learning criterion, a 24-h delay commenced before the second exposure to the test task. (**c**) Conflict: this condition differed from the no-conflict condition, as the training on the FOT was immediately followed by a training on a perceptually overlapping task (POT). Again, after the learning criterion was reached, a 24-h delay commenced before the second exposure to the test task.

To investigate whether any training was prerequisite for solving the test, we introduced (1) a control condition, in which the subject did not receive any trainings. Further, to investigate whether the apes would benefit from a relevant memory in the test, we introduced (2) a no-conflict condition, in which the subject had only the training on the FOT and no conflicting trainings ensued. To investigate whether the subjects would suffer from retrieval competition, we introduced (3) a conflict condition, in which the subject had two trainings, on the FOT and the POT, whose memories would potentially compete for retrieval in the test (see Fig. 1).

All conditions (control, no-conflict, and conflict) began with a baseline trial to assess whether subjects might spontaneously open the test apparatus, prior to training. If their baseline attempts were unsuccessful, subjects advanced to the FOT (in no-conflict and conflict conditions) and POT (only in conflict condition) training apparatuses. Baseline, FOT, and POT trials were conducted in succession, within the same session. The test session was conducted 24 h after the training session (Fig. 1). The baselines included a single trial, contrary to the tests that included multiple trials. For all successful subjects, the tests were slightly longer or much shorter than the baselines (see Table S1).

**Figure 2:**
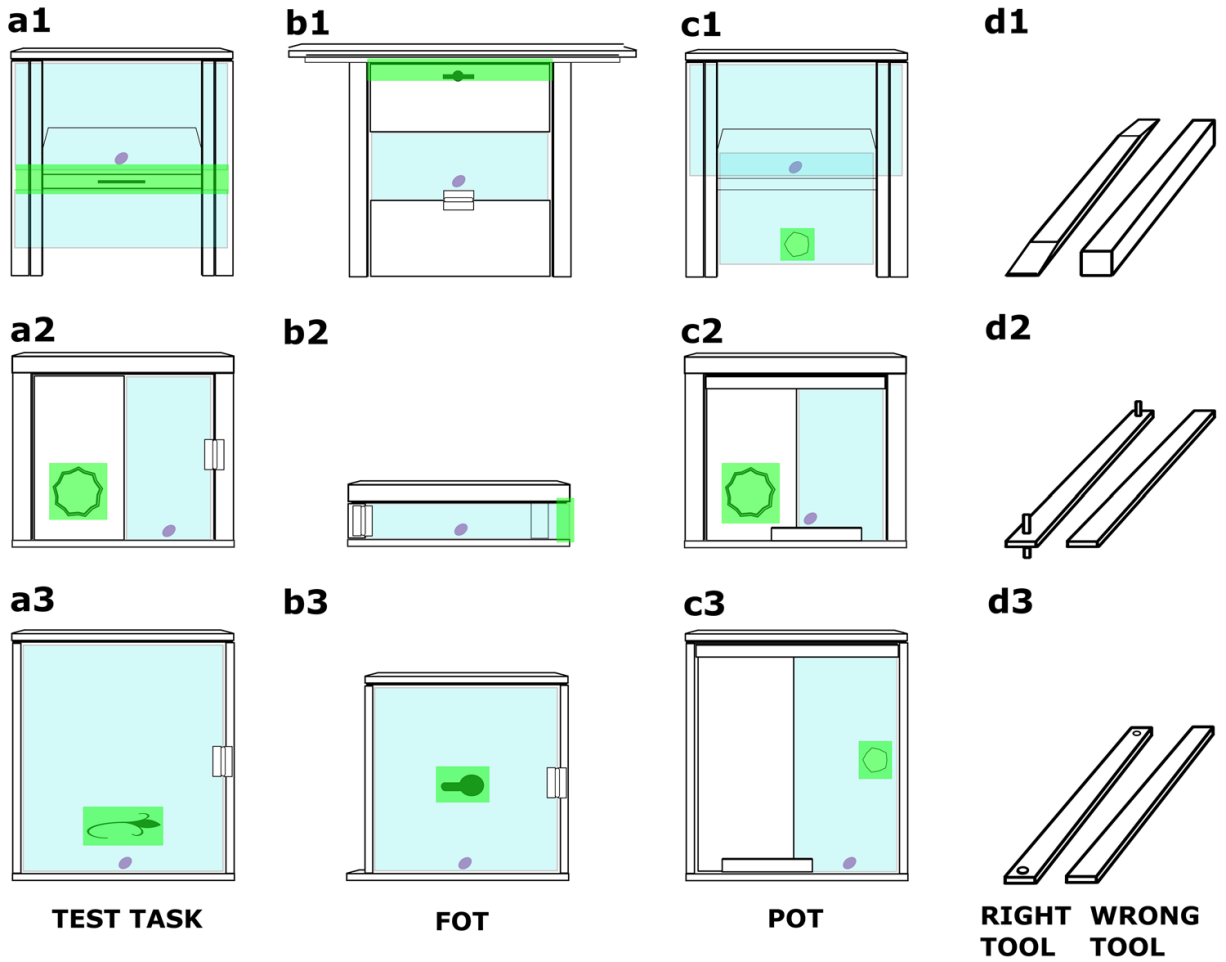
A display of materials: puzzle boxes (**a**–**c**) and tools (**d**) used in the study. The relevant components are highlighted in green; all other components were considered irrelevant. Each set (1–3) consisted of three boxes: a test task (**a**), a functionally overlapping task (FOT; **b**) and a perceptually overlapping task (POT; **c**). The test task and FOT could only be opened with a right tool (**d**, to the left), but POT could be also opened with the wrong tool (**d**, to the right). Once opened, the door either hinged outward toward the subject (in all test tasks and FOTs), or slid upwards/to the side (in all POTs). These rules applied to all three sets. (1) Screwset. (**a1**) The tip of the right tool inserted into the upper gap in the middle part of the front, pushed and lifted. (**b1**) The tip of the right tool inserted horizontally into the upper part of the apparatus, slightly lifted and slowly pulled. (**c1**) The tip of either tool inserted into a hole in the bottom part of the plexiglass door and slid upwards. (2) Hookset. (**a2**) The tip of the right tool inserted into the hole in the front part of the door, hooked and pulled. (**b2**) The tip of the right tool hooked onto the part of the plexiglass door protruding to the right and pulled. (**c2**) The tip of either tool inserted into the hole in the front part of the door and slid to the right. (3) Holeset. (**a3**) The hole in the tip of the right tool cast onto the hook protruding from the door and pulled. (**b3**) The hole in the tip of the right tool cast onto the hook protruding from the door and pulled. (**c3**) The tip of either of the tools inserted into the hole in the plexiglass door and slid to the left.

Each subject completed three sets of puzzle boxes, one in each condition (control, no-conflict, conflict). Each set of puzzle boxes was accompanied by a unique set of tools and had a unique configuration of the relevant/irrelevant components (see Fig. 2 and Fig. S1 online).

### Subjects

Six great apes (1 male orangutan, *Pongo abelii,* 1 male and 4 female chimpanzees, *Pan troglodytes*) participated. They lived with conspecifics at Lund University Primate Research Station Furuvik (Sweden), and had previous experimental experience. Ages varied between 9 and 39 years (see Table 1). They were never food deprived and participated in the tasks voluntarily. Data from all subjects were included in the analyses. The research was approved by the Regional Ethical Review Board at Uppsala District Court (Sweden), permit no. C110/15 and was performed in accordance with relevant guidelines and regulations.

**Table 1:** A list of subjects involved in the study.

Species	Name	Sex	Age	Condition	Set	Score		Test	
						Baseline	Test	Interaction time preceding the 1st relevant interaction [s]	Attempts before success
Sumatran orang-utan *(Pongo abelii)*	Naong	Male	27	Conflict	Holeset	0	1	4.12	2
				No-conflict	Screwset	0	1	0	0
				Control	Hookset	0	0	No relevant interactions	–
chimpanzee *(Pan troglodytes)*	Santino	Male	39	Conflict	Holeset	0	1	31.38	6
				No-conflict	Screwset	0	1	26.915	5
				Control	Hookset	1	–	–	–
	Linda	Female	33	No-conflict	Holeset	0	1	0	0
				Control	Screwset	0	0	No relevant interactions	–
				Conflict	Hookset	0	1	22.98	1
	Selma	Female	9	No-conflict	Holeset	0	1	0	0
				Control	Screwset	1	–	–	–
				Conflict	Hookset	0	1	30.18	1
	Maggan	Female	17	Control	Holeset	0	0	No relevant interactions	–
				Conflict	Screwset	0	1	56.76	1
				No-conflict	Hookset	0	1	4.53	1
	Manda	Female	13	Control	Holeset	0	0	0	–
				Conflict	Screwset	0	0	0.25	–
				No-conflict	Hookset	0	0	3.05	–

Power estimation for binomial outcomes was carried out before testing, based on accuracy mean, inter-individual variation, number of animals, number of trials, number of simulations, significance level and minimum required power (see Supplementary Information 2). However, sample size was predetermined: there were six great apes that could have been tested in this scheme. Although two other apes were housed at the zoo at the time of testing, one, a female orangutan, was tending to a newborn, and the other, a male chimpanzee, was not trained in bartering.

Although the apes had some varying previous testing experience, none of them had participated in a setup that required tool use from behind the cage bars. Further, the apes used sticks as tools on a daily basis within the enclosures, and only two of them (Selma and Naong) were observed to modify the tips.

### Apparatus

Three analogical sets of tasks were devised, each with three puzzle boxes and three tools (see Fig. 2, Fig. S1). All boxes were made from wood with a plexiglass door making the food reward (a grape or a marshmallow) visible. To open a puzzle box, the subject first had to choose a tool and thereafter perform actions on the relevant elements of the box with a tip of the tool (for an example see Fig. S2). The solution always required three actions. The first action involved either of the three: (1) inserting the tip into a gap, (2) hooking the tip behind a surface, (3) casting the tip onto a protruding hook. The second action required stabilizing the hand in a fixed position, and the third action involved either pulling the tool or pushing it to the side/upwards.

The FOT and the test always required the use of the right tool, and the same first and third action, but a different second action (a different position of hand). The POT could have been solved both with the right and the wrong tool, and always required different first, second and third actions than the FOT and the test task. All tools were made of soft wood to avoid injury or damage. To prevent flipping, the boxes were fastened onto a sliding table attached to cage bars which could be moved back and forth by the experimenter. The apes could choose and use tools exclusively from behind the grid patterned bars (4.5 × 4.5 cm) that allowed extending only single digits toward the apparatus.

At least two aspects varied between the sets: (1) the degree of perceptual overlap, and (2) difficulty of required motor patterns. The perceptual overlap between the POT and the test was maximized through identical shapes and dimensions (height, width, length) and a similar distribution of wood and Plexiglas on the front side of the puzzle boxes. The degree of the perceptual overlap varied between the sets, but, as there was only an effect of condition, and not task set on the test score, the degree of the overlap most likely had little effect on the subjects’ performance. However, in the training on the FOT, the holeset required the largest number of demonstrations on the experimenter’s part and the largest number of interactions on the subject’s part before mastering the task (see Table S2).

### Procedure

#### Baselines and tests

The subjects always had unlimited access to all tools available in a given trial, as the tools always lied beside the apparatus within the subjects’ reach. As all apes were proficient at bartering, each tool was retrieved by the end of each trial. Due to tool material choice, at the beginning of trials all subjects but two destroyed the tools, either by biting into the top of the tool or splitting it into smaller wooden pieces, which they subsequently attempted to trade for food items. To avoid impairment of their bartering skills and reinforcement of tool destruction, the experimenter always moved the apparatus away from the subject when it inserted the tool into mouth. Only if the subject stopped this behaviour would the apparatus be moved back. In the test, if the subject destroyed a tool in any way twice in a row, the trial was terminated and qualified as failed.

At baseline, the subjects had a limited time for interaction with the test task, and their first response from taking the tool(s), using them on the apparatus, or destroying or returning them, was recorded. Only a single response was recorded to avoid a prolonged negative (that is not ending with food item’s release) exposure to the task. If the subject carried out a correct action with the functional tool and released the food item at this point, it was excluded from further testing on a given apparatus. This was the case with two apes (Selma and Santino) in the control condition.

In the test, a number of up to 10 attempts, defined as (1) laying the tools out on the apparatus’ tray and moving the apparatus toward the subjects, (2) holding the tray, (3) removing the tray and retrieving the tools, was set as a maximum allowed. The apes could not swap tools within a single attempt; if they did, this was counted as another attempt. For details on individual performance, see Table S1. Otherwise, the apes had unlimited time for interaction with the task, unless they (1) destroyed the tool twice in a row, (2) left the apparatus, or (3) expressed behavioural signs of frustration (e.g., spitting, crossing arms on the chest). These behaviours were mostly evinced during test trials in the control condition, and always led to trial termination (for details see Table S3 online).

Although pre-defined duration of baselines and tests could have been specified, this approach would do less justice to the subjects’ performance than an exploratory approach. Therefore, only the first attempt (defined as above) was recorded at baseline, and the subjects could have interacted with all available tools and the apparatus unless they evinced the above-mentioned behaviours in the test (see Table S3 online). By doing so, unwanted behaviours (tool destruction, leaving the apparatus) were not reinforced, and frustration of the subjects as well as a lack of cooperation with the experimenter in future encounters were minimized.

#### Trainings

During the trainings on the FOT and the POT, the subjects learned how to use the right tool to open the given puzzle box. The training always started with a demonstration by the experimenter, in which she used the right tool to release the food item from the apparatus. The subject always received the released food item and, immediately afterwards, could interact with the apparatus. If the subject did not succeed despite repeated interactions, the experimenter traded the tool for a food item and demonstrated the correct solution again. This sequence was repeated until the subject was able to execute a correct response without the experimenter’s help. If the subject was not able to execute a correct response twice in a row, the experimenter demonstrated the solution again, and this procedure was repeated until the subject was able to release the food item five times in a row. In all cases, once the subject succeeded twice in a row on its own, it did not need further demonstrations to reach the learning criterion. With some subjects, the experimenter was also able to guide the use of the tool by holding the tip of the tool and executing the first action of the motor pattern (hooking, casting, or inserting), which was then completed within the second (adjusting hand position) and the third (pulling or pushing) actions on the subject’s part. Training times were not limited; that is, the subjects could exchange the tools and attempt at solving the FOT and the POT as many times as needed until reaching the learning criterion (see Table S2). However, once the subject reached the learning criterion, the training was terminated.

#### Coding

All trials were video-recorded. For each video, all interactions with the apparatus executed by the subjects were coded frame-by-frame in ELAN 4.9.3. An interaction was defined as a time interval between an onset and an offset of physical contact between a tool held by the subject and an element of the puzzle box used in a given trial. As each interaction involved a certain tool and a certain element of the box, two aspects of the interaction were always determined: (1) the tool used, right (F) or wrong (NF), (2) the component of the apparatus touched, relevant (rel) or irrelevant (irrel).

Two raters coded the videos: one rater coded 100%, and the second rater coded 17.4% of the videos. The second rater downloaded the written instructions and the videos from an online resource without face-to-face contact with the first rater to ensure his independence. Time-unit kappa^44^ was subsequently computed to estimate inter-observer agreement, understood as the accuracy of the overlap between the interval patterns generated by the raters for the same recording. Each of the recordings was divided into consecutive one-second intervals, and for each interval a 0–1 response was determined. Occurrence of coding on the rater’s part was counted as 1, and its lack was counted as 0. The 0–1 responses for each interval were subsequently assembled into a rater-specific pattern, and finally, an inter-rater kappa coefficient was calculated between these two patterns. The agreement was high and equaled 0.995. The analysis was conducted in *R* (v.3.5.1, the R Foundation for Statistical Computing: https://www.R-project.org). Significance level was set at 0.05.

Coding was terminated either with the offset of the last recorded interaction or with the offset of the first interaction that led to food item’s release. For each recording, several variables were computed from the coded intervals. To obtain these variables, certain interaction times were divided either by the overall time between the onset of the first interaction and the offset of the last interaction or by a half, or a fourth of this overall time. For a full list of variables see Table S4.

#### Statistical analysis

A Generalized Linear Mixed Model with Bernoulli distribution was fit to determine the fixed effect of condition on the score (pass/fail; brms package^45,46^, controlling for the random effect of subject ID (control: n = 4, no-conflict: n = 6, conflict: n = 6). A series of Generalized Linear Mixed Models with Dirichlet distribution was used to estimate the fixed effect of condition on proportions of interaction time for specific tools and components of the apparatus in the test (brms package). The Dirichlet analyses were carried out only for those subjects that succeeded in the test. Note that, in the results, the effect sizes’ range equaled [− 200, 200] because two differences in %, each between [− 100, 100], were compared in the analysis.

A Generalized Linear Mixed Model with Bernoulli distribution was fit to determine the fixed effect of task on the score (pass/fail; brms package), controlling for the random effect of subject ID. Times spent on particular trainings (FOT only, or FOT and POT), from the beginning of the first to the end of the last interaction, were used to determine whether such time influenced the success in the test. The duration of the test equaled the overall time of all interactions executed in the test. Generalized Linear Mixed Models with Gamma distribution were used to estimate the effect of times spent on particular trainings and the effect of condition and task on the duration of the test. Further, Generalized Linear Mixed Models with poisson distribution were used to estimate the effect of times spent on particular trainings on the duration of the test. All data was plotted using ggplot2^47^^,^ soundgen^48^ and reshape^49^ packages. For details see the R script in the Supplementary Information 2.

## Results

### Functional overlap promoted success

In the control condition, all subjects that initially failed at baseline also failed to solve the test. In the no-conflict condition, all subjects failed at baseline, and all six proceeded to the training on the FOT. As all subjects but one solved the test task after the training on the FOT, there was a very strong difference in score between the control and the no-conflict condition (100% [97.01–100%]).

### Perceptual overlap did not hinder success

**Figure 3:**
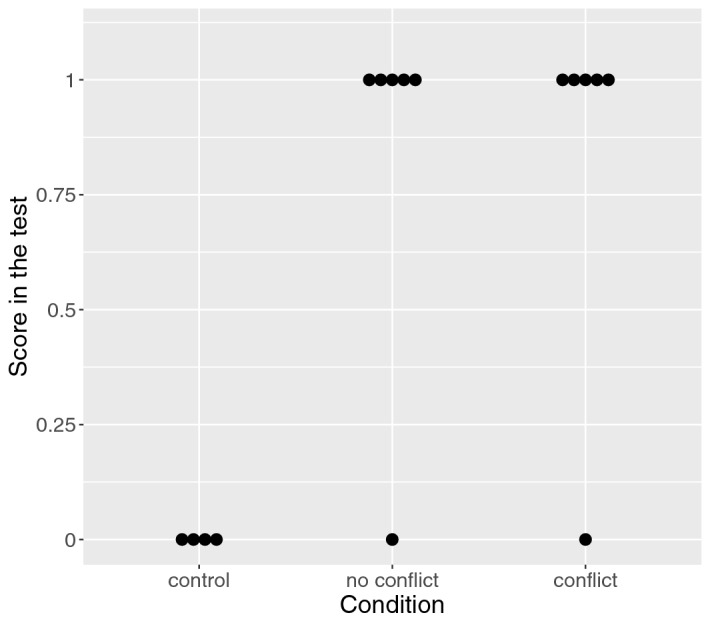
A boxplot of the effect of condition on the score in the test.

In the conflict condition, six subjects proceeded to the trainings on the FOT and the POT and all but one subsequently solved the test (Fig. 3). Notably, the score was higher in the conflict than in the control condition (100% [98.03%, 100%]), but not in the no-conflict condition (0% [− 0.011, 0.013]).

### Functional overlap promoted focusing on the relevant aspects of the problem

The observed differences could be caused by: (1) a benefit of training on the FOT and no cost of the conflicting training on the POT, (2) a benefit of training on the FOT and a cost of the conflicting training on the POT. A further statistical analysis was carried out to determine which effects led to the observed success/failure pattern (see Tables S4, S5 online). We predicted that the apes’ interaction with the test problem would change as a function of time and reflect how memory retrieval affects problem solving (control: no relevant memory; no-conflict: a relevant memory; conflict: a relevant and a conflicting, irrelevant memory). To track these changes, we compared the difference between the relevant and the irrelevant interactions in the first and second half of the test, and in the 1st, 2nd, 3rd and 4th quartile of the test. Since testing whether subjects suffered but eventually resolved the conflict is valid only for these apes that did eventually resolve it, only the successful subjects were involved in this analysis. To establish whether the perceptual overlap hindered solving the test, we also compared the duration of all interactions preceding the first relevant interaction in the test across the conditions. As the subjects received up to 10 attempts at solving the test, its duration varied from 0.1 to 4.6 min between subjects. For plots of proportions of interactions involving a given tool and components to overall interaction time in all test attempts, executed by each of the successful apes, see Fig. S4 online.

In order to assess whether subjects benefited from FOT experience, we compared the time spent on the relevant (right tool + relevant components) and the irrelevant (wrong tool + irrelevant components) interactions in control versus no-conflict and conflict conditions. Recall that only subjects in the control condition did not receive FOT training.

**Figure 4:**
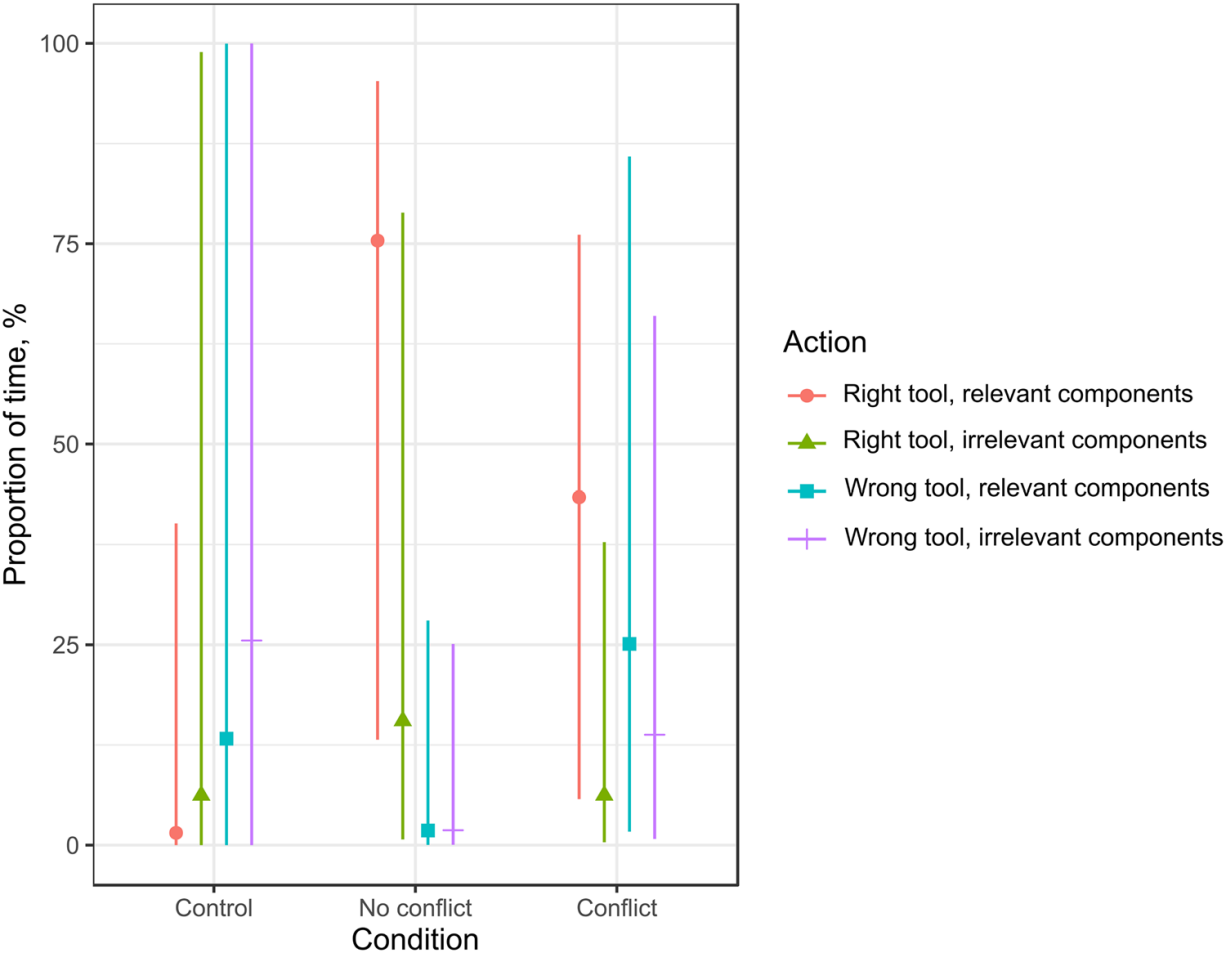
A plot of the effect sizes of condition on all interaction times in the test.

Overall, the apes in the no-conflict and conflict conditions spent more time on the relevant than irrelevant interactions compared to the control condition (no-conflict vs. control: 90.8% [8.7, 185.7]; conflict vs. control: 52.2% [− 34, 151]) and more time on the relevant than all other interactions compared to the control condition (no-conflict vs. control: 93.1% [6.6, 125]; conflict vs. control: 50.6%[− 5.9, 97.5]; see Fig. 4 and Supplementary Table S5). The difference in time spent on the relevant and the irrelevant interactions between the no-conflict and the control condition was strong in both halves of the test (1st half: 61% [− 18.3, 163.6]; 2nd half: 122.5% [55.1, 196.4]). The difference between the conflict and the control condition was weak in the 1st half of the test (0.7% [− 78.9, 100.1) and strong in the 2nd half of the test (80.4% [1.2, 164.3]). The analysis of such differences across quartiles of the test revealed that shifts in the difference between the relevant and the irrelevant interactions had different dynamics across conditions. With regards to the no-conflict versus control condition, the difference between the relevant and the irrelevant interactions was strong already in the 1st quartile (46.8% [− 22.5, 141.9]), and became stronger in each of the next quartiles, with 64.1% [− 9.1, 164.9] in the 2nd quartile, 80.5% [7, 174.2] in the 3rd quartile and 144.7% [65.8, 196.6] in the 4th quartile. With regards to the conflict versus control condition, the difference between the relevant and the irrelevant interactions was very weak in the 1st quartile (0.2% [− 64, 91.5]), and became slightly stronger in the 2nd and the 3rd quartile (8.9% [− 52.2, 47.1] and 15% [− 29.5, 53], respectively), and very strong in the 4th quartile (100.5% [38.4, 122.4]).

In the control conditions, the apes that succeeded in other conditions did not execute a single relevant interaction in the test.

### Perceptual overlap hindered focusing on the relevant aspects of the problem

In order to assess whether subjects were hindered by POT experience, we compared differences between time spent on relevant and irrelevant interactions in the no-conflict vs. conflict conditions. Recall that only subjects in the conflict condition received the POT training. Overall, the apes in the no-conflict condition spent more time on the relevant than irrelevant interactions compared to the conflict condition (41.5% [− 20.4, 109.5]) and more time on the relevant than all other interactions compared to the conflict condition (38.5% [− 33.8, 95,9]; see Fig. 4 and Supplementary Table S5). The difference in time spent on the relevant and the irrelevant interactions between the no-conflict and the conflict condition was weaker in the 2nd than in the 1st half of the test (1st half: 59.2% [− 1.6, 127.5]; 2nd half: 42.1% [7.6, 97]). The analysis of this difference across quartiles of the test revealed that the difference slightly increased in the first three quartiles (1st quartile: 45.8% [− 8.9, 102.1]; 2nd quartile: 57.2% [2.9, 109.8]; 3rd quartile: 61.9% [5.9, 124.2]) and then dramatically decreased in the 4th quartile (16.8% [0.8, 51]). Further, the apes took significantly less time to execute the first relevant interaction in the no-conflict than in the conflict condition (z = − 2.544, p = 0.011).

### Time spent on the trainings did not influence performance in the test

The success in the conflict condition could be explained by a stronger memory trace of the training on the FOT; if the apes spent more time on this training than on the training on the POT, this explanation could be valid. However, in the conflict condition there was no effect of the proportion of time spent on either of the tasks (FOT or POT) to overall training time on the test score (Chi(1) = 0.213, p = 0.645). Out of five subjects that solved the test, two spent more time interacting with the POT than the FOT, while three spent more time interacting with the FOT than the POT.

The duration of the FOT training varied between subjects, from 5 to 77 min, but there was no effect of the duration of the FOT training on the duration of the test (Chi(1) = 1.215, p = 0.27). Likewise, there was no effect of the duration of the POT on the duration of the test (Chi(1) = 3.246, p = 0.072). There was also no effect of the duration of the FOT on the duration of the POT (Chi(1) = 0.546, p = 0.46). The number of attempts at solving the test was also independent from the duration of the FOT (Chi(1) = 0.098, p = 0.757) and the duration of the POT (Chi(1) = 0.333, p = 0.564).

Although three different task sets were introduced, the subjects’ scores did not depend on the task set assigned in the test, as differences in score between the tasks were very weak (holeset vs. hookset: 0.2% [− 54.3, 59.8]; holeset vs. screwset: 6% [− 55.95%, 61.43%]; hookset vs. screwset: 0% [− 59.17%, 57.49%]). In the successful subjects, solving the holeset took significantly longer in the conflict condition than the no-conflict condition (z = − 3.486, p = 0.006), but the difference between conditions was not significant for the other task sets (hookset: z = − 0.592, p = 0.991; screwset: z = − 0.07, p = 1).

### The apes struggled with, but resolved retrieval competition

Confronted with the test task, the apes had to use any past learning experiences that could inform response to the present situation. Ideally, they should target and retrieve those features of the past situations that were somewhat relevant for the situation at hand; and mapping of the relevant features onto the present situation should result in focusing on the relevant components and/or the right tool, at the cost of the irrelevant components and/or the wrong tool.

**Figure 5:**
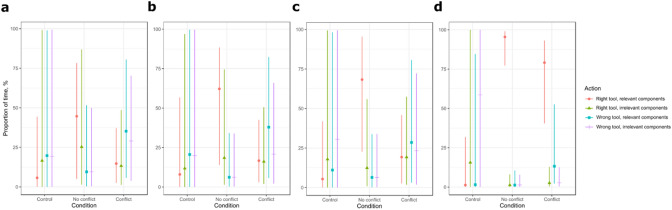
A plot of the effect sizes of condition on interaction times throughout the test: (**a**) in the 1st quartile, (**b**) in the 2nd quartile, (**c**) in the 3rd quartile, (**d**) in the 4th quartile. For plots of proportions of given interaction times to overall interaction time in all test attempts, executed by each of the successful apes, see Fig. S4.

The pattern of interactions between the right/wrong tool and the relevant/irrelevant components differed between the three conditions (Fig. 5 and Fig. S4 online), revealing what may have happened in the apes’ memory in the test.

Each condition presented a unique challenge to the apes. In the conflict condition, the apes had to overcome the cueing to the recent yet irrelevant memory; in the no-conflict condition, the apes had to generalise the previously acquired experience onto a new situation; and in the control condition, the apes had no new memories to act upon in the test. However, some of the apes may have had an edge over others, depending on the available previous experiences, as the proportion of the relevant interactions to overall interaction time was highest in the no-conflict condition, lower for the conflict condition and lowest for the control condition (for details see “Perceptual overlap hindered focusing on the relevant aspects of the solution” and Table S5).

Having a functionally overlapping memory was already an advantage at the start, as in the 1st quartile the apes in the no-conflict condition spent much more time on interacting with the right tool and the relevant components than in the other conditions. At the beginning of the test, having a conflicting, perceptually overlapping memory was a strong disadvantage. Even though, in the 1st quartile, the apes in the conflict condition interacted more with the right tool and the relevant components than the apes in the control condition, they also interacted more with the wrong tool and the irrelevant components than in the control condition. They also interacted more with the right tool and the irrelevant components than in the control and the no-conflict conditions. This is not surprising, as only then did they have a memory of using that tool for interacting with the relevant aspects of the perceptually overlapping situation; as that memory competed for retrieval with the functionally overlapping memory, it mapped onto the present situation and elicited those interactions.

In the 2nd quartile, the apes in the conflict condition still interacted most with the right tool and the irrelevant components of all conditions but noted a drop in interactions with the wrong tool and the irrelevant components. A similar drop occurred for the right tool and the irrelevant components in the 3rd quartile, in which the apes also started to interact more with the right tool and the relevant components than in the control condition, but still much less than in the no-conflict condition. This changed in the 4th quartile, in which the apes in the conflict condition shifted their attention toward the right tool and the relevant components, noting a considerable spike in such interactions. This spike was independent from the last action, leading directly to solving the test, as the size effects reported in Table S5 show.

Even in the 4th quartile, in which the apes in the conflict condition focused considerably more on the right tool and the relevant components than before, this focus was much smaller than in the no-conflict condition. This suggests that, even soon before solving the test, the apes with the perceptually overlapping memory were less attentive to the relevant aspects of the problem and its solution than those that only had the functionally overlapping memory.

## Discussion

The results suggest that great apes are able to solve problems by using, and sorting between, memory traces of similar experiences. Overall, the apes attended to the relevant aspects of the problems to a greater extent in both the no-conflict and the conflict conditions than in the control condition.

The apes suffered from retrieval competition but overcame the memory interference as they eventually succeeded in solving the problem. Having conflicting memories of functional and perceptual overlaps resulted in a different performance than having only a functionally overlapping memory. Before eventually solving the problem, the apes with conflicting memories attended less to the relevant aspects of the apparatus and were less likely to use an appropriate solution. The only ape that failed to solve the problem with conflicting memories also failed when it had only a non-conflicting one. In the future, this result could be replicated with a larger population of great apes. As we tested only six individuals of two species it is difficult to generalize our results onto the general population of chimpanzees and orangutans, and onto other great ape species. While we showed that five great apes were capable of resolving conflicts between memories and only one was not, this 5:1 ratio may differ in the general population and between great ape species. It is noteworthy that two apes managed to spontaneously solve the task at baseline, showing that at least some individuals could succeed without a previous functionally overlapping experience. In future studies, more motorically challenging tasks could also be used to prevent the apes from solving them at baseline.

The current experimental paradigm was set up to boost the salience of irrelevant, competing memories through the manipulation of recency, perceptual overlap and prior tool-use. The irrelevant, perceptually overlapping memory trace was more recent and more similar than the relevant one, and the wrong tool was involved in the perceptually overlapping, but not in the functionally overlapping training. Together, these factors caused interference due to conflict between memories for previous learning experiences with predicted impairments in goal-relevant task performance. We posit that manipulating the levels of these factors would be desired in future research and is critical to disentangling the relative contributions of recency, perceptual overlap and tool experience to memory conflict resolution in great apes.

This study shows that great apes can overcome competing, irrelevant memory traces in favour of relevant ones when facing a novel problem where the perceptual similarity cannot inform the functional relevance. This indicates that great apes’ cognitive executive function repertoire includes resolution of memory competition. Suffering from, yet resolving, memory interference is considered a hallmark of flexible retrieval in humans^50,51^. Interference results from retrieval competition that occurs when two memories, a relevant and an irrelevant one, are cued by the present retrieval situation. Such interference triggers executive control mechanisms that resolve the resulting memory conflict by selectively retrieving the relevant target memory^52,57^. While previous studies have revealed that chimpanzees could, to some extent, resolve interference in a set-shifting task, where relevant and perceptually salient information lost its relevance within a test^53^ and in a working-memory task^54^^,^ our study provides novel evidence of great apes’ capacity for interference resolution in long-term memory. Future studies could address the links between working memory and long-term memory conflict resolution in great apes.

Our results also suggest that great apes actively recognize which memories are relevant for the task at hand. In the test situation, the apes were likely first cued to the recent memory trace of the perceptually similar apparatus. However, they overcame this misrepresentation and instead relied on a previous memory that corresponded to the function of the apparatus and solved the task more readily than when they had no related previous experience. If the apes were unable to re-connect to a relevant memory, they would have been stuck with the memory that was first cued and would not have been able to solve the task in the flexible way they did. These results may indicate that great apes are capable of some form of representation of the connection between their memories and reality, or that their affective systems relating to memories are well-developed.

The levels of innovation in great apes are among the highest of all studied animals, and it has been argued that innovation is proximately brought about by some sort of behavioural flexibility in combination with exploration^18^. We suggest that it is possible that the ability to sort between memory traces and overcome conflict from irrelevant ones is one of the central cognitive mechanisms behind technical innovation. It warrants a flexible application of previous experiences to novel problems. This executive function may also explain why great apes are capable of flexible planning, and, for example, select tools they have never used before for a future situation and produce appropriate tools for future use^55^.

In humans, competition resolution mediating selective retrieval of the most relevant memory comes at a cost: it spurs retrieval-induced forgetting^56^. Inhibitory control mechanisms are recruited to handle interference from competing memories by lowering their level of activation, and this results in forgetting of the competitors^7,57–59^. Such forgetting is adaptive because it reduces the likelihood of future competition as the irrelevant memories gradually fade away^60,61^. Bekinschtein and colleagues recently showed prefrontal cortex-dependent retrieval-induced forgetting in rats, with effects and mechanisms similar to those in humans^51^. The set-up we used in this study could be further adapted to also include tests of progressive forgetting. A shift of focus towards forgetting as an adaptive function of memory flexibility could shed more light on the evolution of memory mechanisms.

As the executive function that inhibits irrelevant memory traces may be responsible for a range of flexible behaviours, we think that a more standardized test should be developed. This would help in studying various species in a comparative way. Results from such tests could be correlated with neurobiological factors, ecology and performance in other cognitive tests, and reveal the broader significance and evolution of this executive function. It might be more illuminating to study memory flexibility, rather than accuracy, in order to understand the evolution of complex cognition.

### Ethical approval

All experimental protocols were approved by the Regional Ethical Review Board at Uppsala District Court (Sweden), permit no. C110/15 and were performed in accordance with relevant guidelines and regulations.

## Supplementary information

Supplementary file 1

Supplementary file 2

Supplementary file 3

## Data Availability

All data generated or analysed during this study are included in this published article and its Supplementary Information files.
