# Supplementary material for: Great apes selectively retrieve relevant memories to guide action
Source: Sci Rep. 2020 Jul 28;10:12603. doi: 10.1038/s41598-020-69607-6 (PMC7387339; doi:10.1038/s41598-020-69607-6)
Supplement: Supplementary file 1 [file 41598_2020_69607_MOESM1_ESM.docx]

Great apes selectively retrieve relevant memories
to guide action

Katarzyna Bobrowicz^1,2*^, Mikael Johansson^2^, Mathias Osvath^1^

^1^ Department of Philosophy and Cognitive Science, Lund University, Lund, Sweden

^2^ Department of Psychology, Lund University, Lund, Sweden

*katarzyna.bobrowicz@gmail.com

**Supplementary Figures**


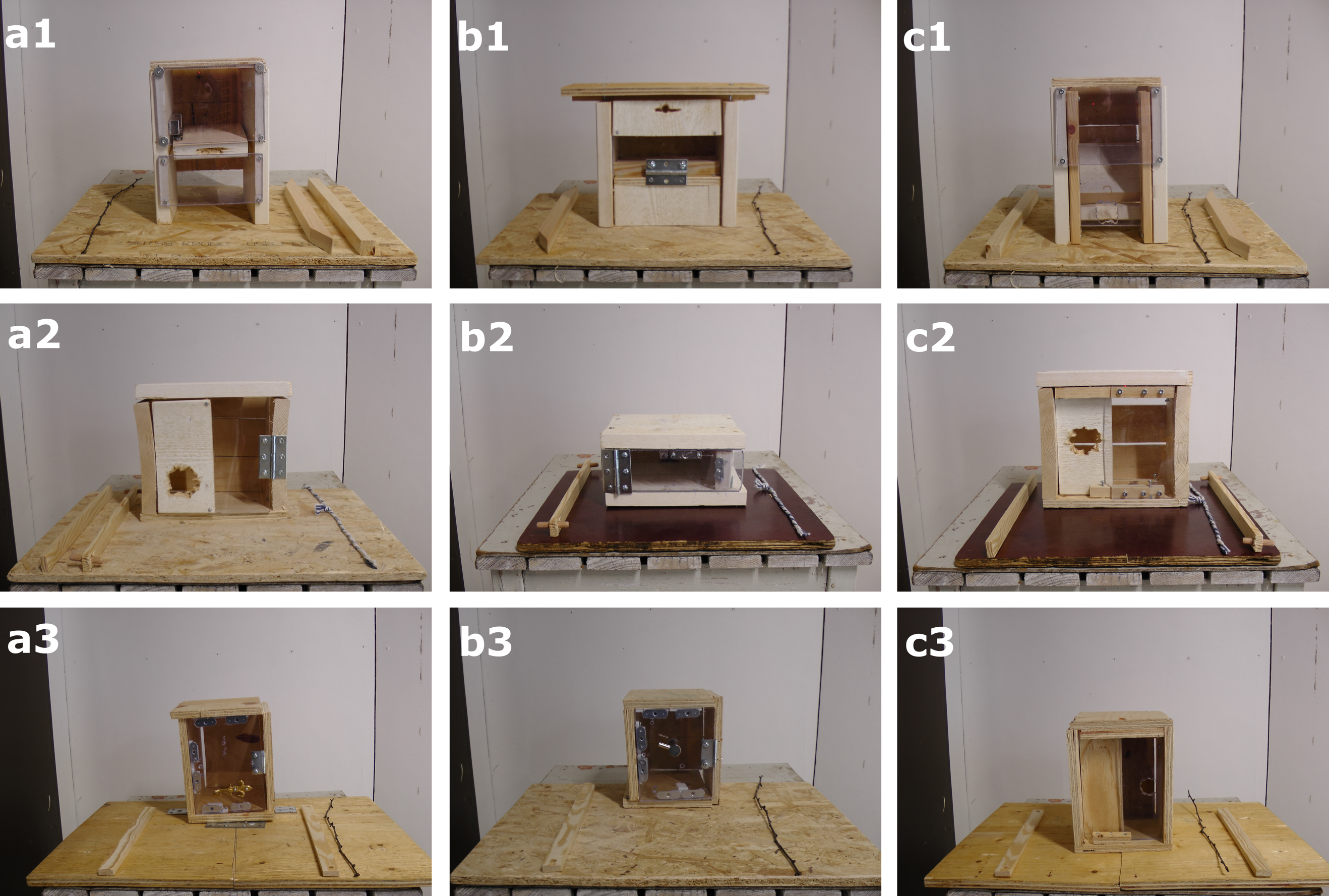


**Fig. S1.** **Photos of the puzzle boxes and the corresponding tools.** The labels from Figure 2 were preserved for reference. (1a-c) Screwset. (2a-c) Hookset. (3a-c) Holeset. POTs (c1-3) and test tasks (a1-3) were defined as perceptually overlapping as their height, width and length were identical. The perceptual overlap between boxes was maximized through a similar distribution of wood and Plexiglas on the front side of the puzzle boxes. The degree of the perceptual overlap varied between the sets, but it did not affect the score in the test.


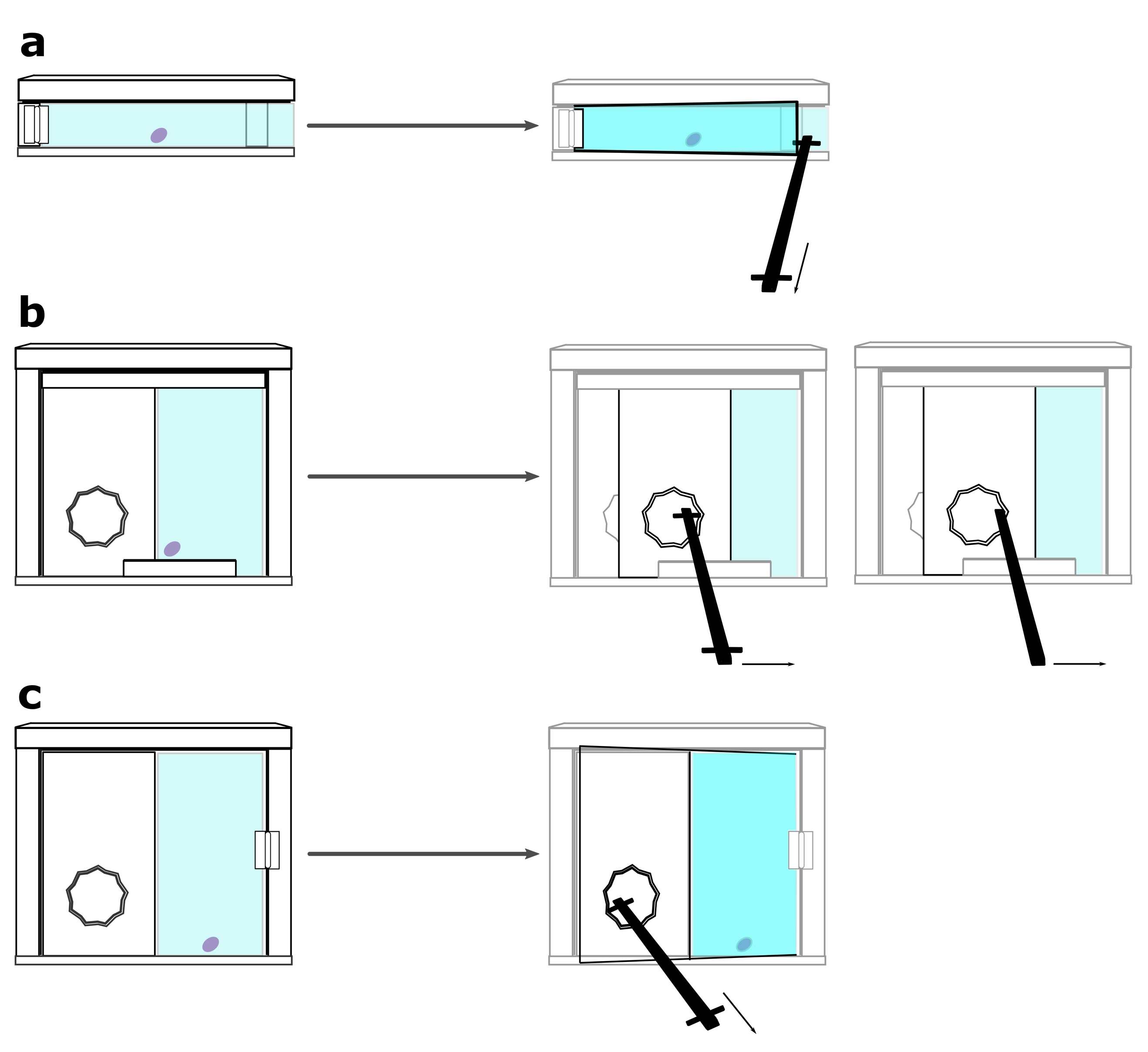


**Fig. S2.** **A display of solutions to each of the hookset tasks**. (a) The functionally overlapping task (FOT) required the right tool. One had to place either of its functional tips behind a part of plexiglass door protruding to the right and then pull the tool to open the door. (b) The perceptually overlapping task (POT) could be opened both with the right and the wrong tool. One had to insert the tip of the tool into the irregular hole in the front of the apparatus and push the tool to the right to open the door. (c) The test task required the right tool; one had to insert the functional tip into the irregular hole in the front of the apparatus and pull the tool to open the door.


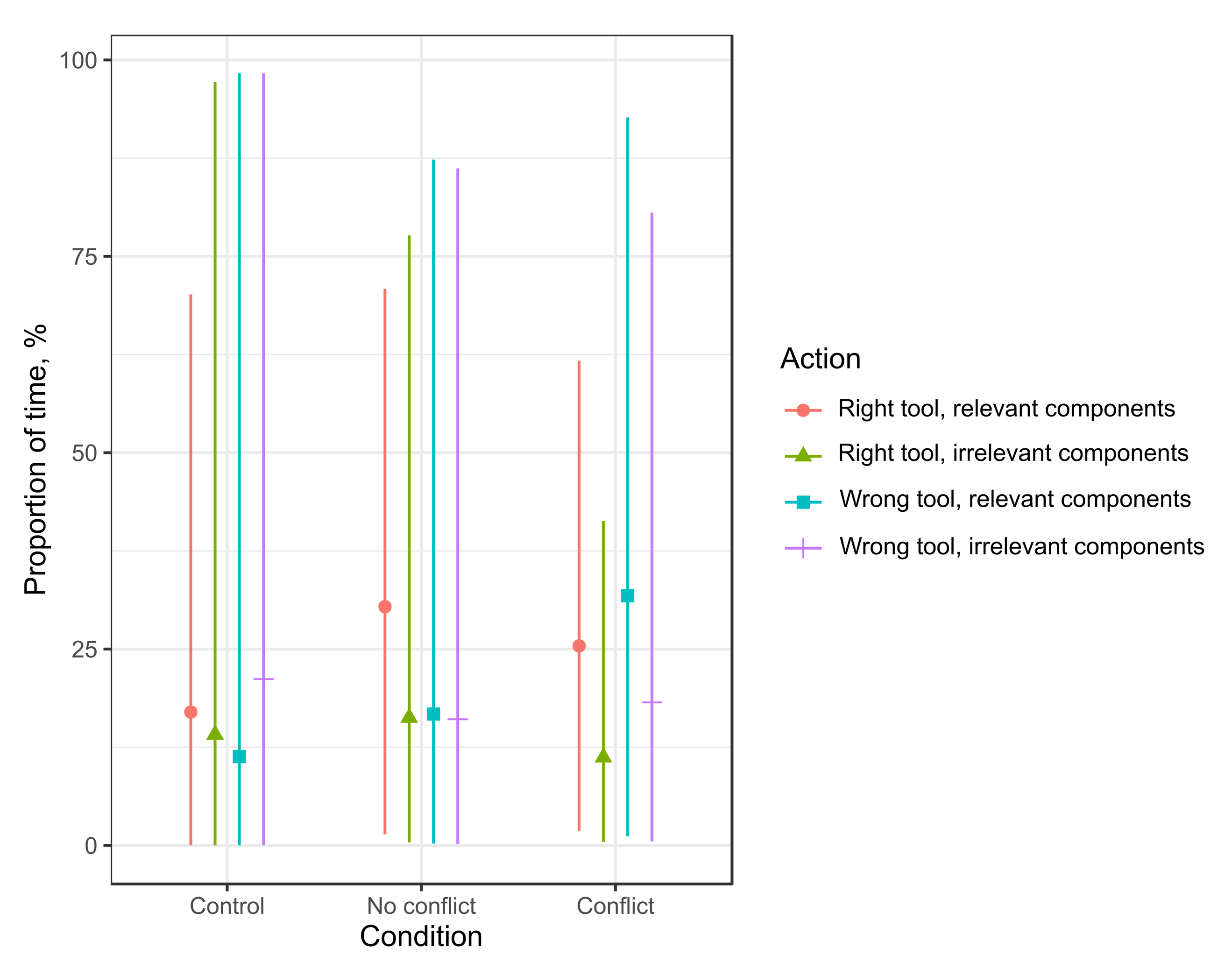


**Fig. S3.** **A plot of the effect sizes of condition on all interaction times in the baseline**. For the analogical plot for the test, see Figure 4 in the main text.


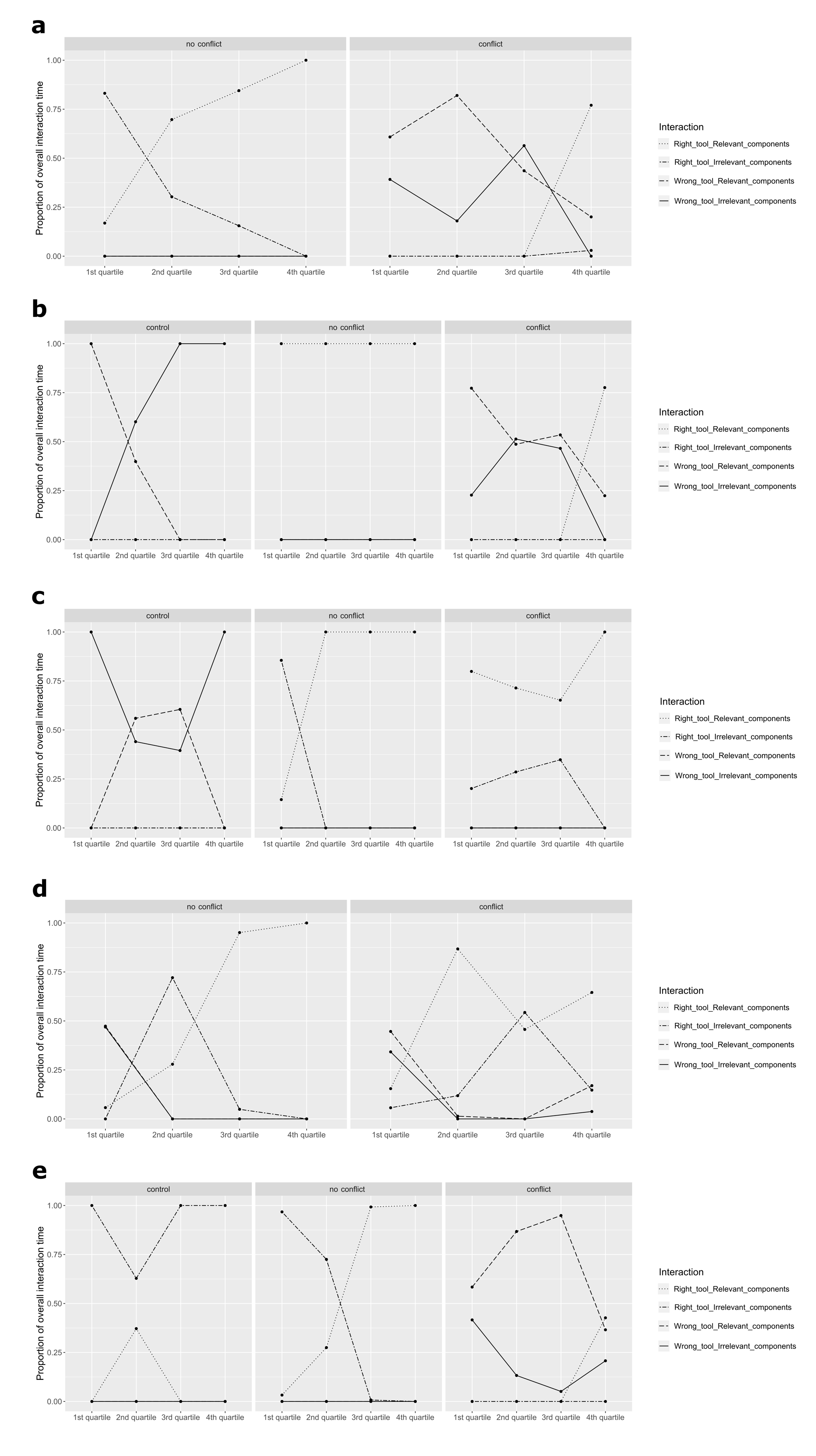


**Fig. S4.** **A plot of proportions of given interactions to overall interaction time in the test, executed by each of the successful apes: (a) Selma, (b) Linda, (c) Naong, (d) Santino, (e) Maggan.** Note that Selma and Santino solved the test task already at baseline and, therefore, did not proceed to the control.

**Supplementary Tables**

**Table S1.** **Parameters of performance by individual, set and condition**. 1^st^ tool - the first tool chosen by the subject; right – the right tool; wrong – the wrong tool; useless – the useless tool; both – the right and the wrong tool; none – none of the tools; TD – tool destruction; time – overall time in minutes from touching one of the tools by the subject to removing the tray by the experimenter. For the test, a combined time from all attempts is displayed, and delays between separate attempts were removed from the overall time.

| **Name** | **Set** | **Condition** | **Baseline** | | | | **Test** | | | | |
| --- | --- | --- | --- | --- | --- | --- | --- | --- | --- | --- | --- |
|  |  |  | **1^st^ tool** | **tools used** | **TD** | **time** | **1^st^ tool** | **tools used** | **TD** | **time** | **attempts  before success** |
| Naong | holeset | conflict | wrong | both | yes | 4.02 | right | wrong | yes | 4.13 | 2 |
|  | screwset | no-conflict | wrong | both | yes | 2.12 | right | both | no | 0.281 | 0 |
|  | hookset | control | wrong | both | yes | 1.49 | wrong | wrong | yes | 1.6 | - |
| Santino | holeset | conflict | right | right | no | 1.2 | wrong | both | no | 4.6 | 6 |
|  | screwset | no-conflict | useless | useless | no | 1.53 | wrong | both | no | 2.1 | 5 |
|  | hookset | control | right | right | no | - | - | - | - | - | - |
| Linda | holeset | no-conflict | wrong | wrong | no | 0.15 | wrong | both | no | 0.11 | 0 |
|  | screwset | control | wrong | wrong | no | 0.85 | wrong | wrong | no | 0.42 | - |
|  | hookset | conflict | wrong | wrong | no | 0.92 | right | right | no | 0.65 | 1 |
| Selma | holeset | no-conflict | right | right | no | 1.3 | right | right | no | 0.59 | 0 |
|  | screwset | control | wrong | both | no | - | - | - | - | - | - |
|  | hookset | conflict | wrong | wrong | no | 1.38 | wrong | both | no | 0.61 | 1 |
| Maggan | holeset | control | right | right | yes | 0.4 | right | right | yes | 1.25 | - |
|  | screwset | conflict | none | none | yes | 0.9 | wrong | both | yes | 1.85 | 1 |
|  | hookset | no-conflict | right | right | yes | 1.46 | right | right | no | 0.9 | 1 |
| Manda | holeset | control | right | both | yes | 1.412 | right | both | yes | 1.46 | - |
|  | screwset | conflict | wrong | both | yes | 2.34 | right | both | yes | 4.47 | - |
|  | hookset | no-conflict | wrong | wrong | no | 1.11 | right | both | yes | 5.25 | - |

**Table S2.** **An overview of the raw data from trainings on the FOT and the POT**: (1) experimenter’s demonstrations, (2) subject’s interactions with an apparatus, before the first success of five consecutive food item releases and reaching the learning criterion.

| **Training task** | **Set name** | **Subject** | **Demonstrations** | **Interactions** |
| --- | --- | --- | --- | --- |
| FOT | holeset | Selma | 56 | 299 |
|  |  | Linda | 11 | 85 |
|  | hookset | Manda | 8 | 316 |
|  |  | Maggan | 23 | 70 |
|  | screwset | Naong | 13 | 10 |
|  |  | Santino | 10 | 28 |
| POT | holeset | Naong | 15 | 32 |
|  |  | Santino | 68 | 317 |
|  | hookset | Selma | 1 | 6 |
|  |  | Linda | 1 | 1 |
|  | screwset | Maggan | 25 | 34 |
|  |  | Manda | 21 | 87 |

**Table S3.** **Behavioural reasons for termination of the second exposure.** Selma and Santino are missing because they succeeded at baseline.

| **No.** | **Name** | **Condition** | **Behavioral reason for termination** |
| --- | --- | --- | --- |
| 1 | Naong | control | tool destruction, crossing arms on the chest |
| 2 | Maggan | control | tool destruction (twice) |
| 3 | Linda | control | spitting |
| 4 | Manda | control | tool destruction (twice) |
| 5 | Manda | conflict | tool destruction (twice) |
| 6 | Manda | no-conflict | tool destruction (twice) |

**Table S4.** **Equations for the interaction variables**. X stands for a fourth of the overall interaction time in the test, all attempts included. Each letter represents time spent on certain interactions in [s].

| **No.** | **Variable** | | | **Equation** | **Definition** |
| --- | --- | --- | --- | --- | --- |
|  | **tools** | **components** | **time** |  |  |
| **1** | right | relevant | overall | (a+e+i+m)/4X | a proportion of time spent on interactions between a right tool and relevant components of an apparatus to overall time spent on interactions between all tools and all components of the apparatus |
| **2** | right | irrelevant | overall | (b+f+j+n)/4X | a proportion of time spent on interactions between a right tool and irrelevant components of an apparatus to overall time spent on interactions between all tools and all components of the apparatus |
| **3** | wrong | relevant | overall | (c+g+k+o)/4X | a proportion of time spent on interactions between a wrong tool and relevant components of an apparatus to overall time spent on interactions between all tools and all components of the apparatus |
| **4** | wrong | irrelevant | overall | (d+h+l+p)/4X | a proportion of time spent on interactions between a wrong tool and irrelevant components of an apparatus to overall time spent on interactions between all tools and all components of the apparatus |
| **5** | right | relevant | 1st half | (a+e)/2X | a proportion of time spent on interactions between a right tool and relevant components of an apparatus to overall time spent on interactions between all tools and all components of the apparatus in the first half of a test/a baseline |
| **6** | right | irrelevant | 1st half | (b+f)/2X | a proportion of time spent on interactions between a right tool and irrelevant components of an apparatus to overall time spent on interactions between all tools and all components of the apparatus in the first half of a test/a baseline |
| **7** | wrong | relevant | 1st half | (c+g)/2X | a proportion of time spent on interactions between a wrong tool and relevant components of an apparatus to overall time spent on interactions between all tools and all components of the apparatus in the first half of a test/a baseline |
| **8** | wrong | irrelevant | 1st half | (d+h)/2X | a proportion of time spent on interactions between a wrong tool and irrelevant components of an apparatus to overall time spent on interactions between all tools and all components of the apparatus in the first half of a test/a baseline |
| **9** | right | relevant | 2nd half | (i+m)/2X | a proportion of time spent on interactions between a right tool and relevant components of an apparatus to overall time spent on interactions between all tools and all components of the apparatus in the second half of a test/a baseline |
| **10** | right | irrelevant | 2nd half | (j+n)/2X | a proportion of time spent on interactions between a right tool and irrelevant components of an apparatus to overall time spent on interactions between all tools and all components of the apparatus in the second half of a test/a baseline |
| **11** | wrong | relevant | 2nd half | (k+o)/2X | a proportion of time spent on interactions between a wrong tool and relevant components of an apparatus to overall time spent on interactions between all tools and all components of the apparatus in the second half of a test/a baseline |
| **12** | wrong | irrelevant | 2nd half | (l+p)/2X | a proportion of time spent on interactions between a wrong tool and irrelevant components of an apparatus to overall time spent on interactions between all tools and all components of the apparatus in the second half of a test/a baseline |
| **13** | right | relevant | 1st quartile | a/X | a proportion of time spent on interactions between a right tool and relevant components of an apparatus to overall time spent on interactions between all tools and all components of the apparatus in the 1st quartile of a test |
| **14** | right | irrelevant | 1st quartile | b/X | a proportion of time spent on interactions between a right tool and irrelevant components of an apparatus to overall time spent on interactions between all tools and all components of the apparatus in the 1st quartile of a test |
| **15** | wrong | relevant | 1st quartile | c/X | a proportion of time spent on interactions between a wrong tool and relevant components of an apparatus to overall time spent on interactions between all tools and all components of the apparatus in the 1st quartile of a test |
| **16** | wrong | irrelevant | 1st quartile | d/X | a proportion of time spent on interactions between a wrong tool and irrelevant components of an apparatus to overall time spent on interactions between all tools and all components of the apparatus in the 1st quartile of a test |
| **17** | right | relevant | 2nd quartile | e/X | a proportion of time spent on interactions between a right tool and relevant components of an apparatus to overall time spent on interactions between all tools and all components of the apparatus in the 2nd quartile of a test |
| **18** | right | irrelevant | 2nd quartile | f/X | a proportion of time spent on interactions between a right tool and irrelevant components of an apparatus to overall time spent on interactions between all tools and all components of the apparatus in the 2nd quartile of a test |
| **19** | wrong | relevant | 2nd quartile | g/X | a proportion of time spent on interactions between a wrong tool and relevant components of an apparatus to overall time spent on interactions between all tools and all components of the apparatus in the 2nd quartile of a test |
| **20** | wrong | irrelevant | 2nd quartile | h/X | a proportion of time spent on interactions between a wrong tool and irrelevant components of an apparatus to overall time spent on interactions between all tools and all components of the apparatus in the 2nd quartile of a test |
| **21** | right | relevant | 3rd quartile | i/X | a proportion of time spent on interactions between a right tool and relevant components of an apparatus to overall time spent on interactions between all tools and all components of the apparatus in the 3rd quartile of a test |
| **22** | right | irrelevant | 3rd quartile | j/X | a proportion of time spent on interactions between a right tool and irrelevant components of an apparatus to overall time spent on interactions between all tools and all components of the apparatus in the 3rd quartile of a test |
| **23** | wrong | relevant | 3rd quartile | k/X | a proportion of time spent on interactions between a wrong tool and relevant components of an apparatus to overall time spent on interactions between all tools and all components of the apparatus in the 3rd quartile of a test |
| **24** | wrong | irrelevant | 3rd quartile | l/X | a proportion of time spent on interactions between a wrong tool and irrelevant components of an apparatus to overall time spent on interactions between all tools and all components of the apparatus in the 3rd quartile of a test |
| **25** | right | relevant | 4th quartile | m/X | a proportion of time spent on interactions between a right tool and relevant components of an apparatus to overall time spent on interactions between all tools and all components of the apparatus in the 4th quartile of a test |
| **26** | right | irrelevant | 4th quartile | n/X | a proportion of time spent on interactions between a right tool and irrelevant components of an apparatus to overall time spent on interactions between all tools and all components of the apparatus in the 4th quartile of a test |
| **27** | wrong | relevant | 4th quartile | o/X | a proportion of time spent on interactions between a wrong tool and relevant components of an apparatus to overall time spent on interactions between all tools and all components of the apparatus in the 4th quartile of a test |
| **28** | wrong | irrelevant | 4th quartile | p/X | a proportion of time spent on interactions between a wrong tool and irrelevant components of an apparatus to overall time spent on interactions between all tools and all components of the apparatus in the 4th quartile of a test |

**Table S5. An overview of effects sizes and confidence intervals for each pair of conditions and two pairs of interactions, used in the manuscript.** Note that the effect sizes’ range equals [-200, 200] because two differences in %, each between [-100, 100] were compared in the analysis.

| Test span | Test time | Conditions | Interactions | Effect size [%] | CI |
| --- | --- | --- | --- | --- | --- |
| All interactions executed during all test attempts | overall time | conflict vs. control | F_rel vs. NF_irrel | 52.2 | [-34, 151] |
|  |  | no conflict vs. control | F_rel vs. NF_irrel | 90.8 | [8.7, 185.7] |
|  |  | conflict vs no conflict | F_rel vs. NF_irrel | 41.5 | [-20.4, 109.5] |
|  |  | conflict vs. control | F_rel vs. all | 50.6 | [-5.9, 97.5] |
|  |  | no conflict vs. control | F_rel vs all | 93.1 | [6.6, 125] |
|  |  | conflict vs no conflict | F_rel vs. all | 38.5 | [-33.8, 95.9] |
|  | 1st half | conflict vs. control | F_rel vs. NF_irrel | 0.7 | [-78.9, 100.1] |
|  |  | no conflict vs. control | F_rel vs. NF_irrel | 61 | [-18.3, 163.6] |
|  |  | conflict vs no conflict | F_rel vs. NF_irrel | 59.2 | [-1.6, 127.5] |
|  |  | conflict vs. control | F_rel vs. all | 6.2 | [-52.2, 44.7] |
|  |  | no conflict vs. control | F_rel vs all | 56.8 | [-16.5, 110.6] |
|  |  | conflict vs no conflict | F_rel vs. all | 51.1 | [-10.8, 103] |
|  | 2nd half | conflict vs. control | F_rel vs. NF_irrel | 80.4 | [1.2, 164.3] |
|  |  | no conflict vs. control | F_rel vs. NF_irrel | 122.5 | [55.1, 196.4] |
|  |  | conflict vs no conflict | F_rel vs. NF_irrel | 42.1 | [7.6, 97] |
|  |  | conflict vs. control | F_rel vs. all | 73.2 | [10.5, 109.7] |
|  |  | no conflict vs. control | F_rel vs all | 120.6 | [58.7, 131.9] |
|  |  | conflict vs no conflict | F_rel vs. all | 43.1 | [5.9, 96.5] |
|  | 1st quartile | conflict vs. control | F_rel vs. NF_irrel | 0.2 | [-64, 91.5] |
|  |  | no conflict vs. control | F_rel vs. NF_irrel | 46.8 | [-22.5, 141.9] |
|  |  | conflict vs no conflict | F_rel vs. NF_irrel | 45.8 | [-8.9, 102.1] |
|  |  | conflict vs. control | F_rel vs. all | 9.4 | [-37.9, 41.1] |
|  |  | no conflict vs. control | F_rel vs all | 46.4 | [-14.2, 96.9] |
|  |  | conflict vs no conflict | F_rel vs. all | 37.6 | [-13.7, 86.2] |
|  | 2nd quartile | conflict vs. control | F_rel vs. NF_irrel | 6.4 | [-63.2, 103.4] |
|  |  | no conflict vs. control | F_rel vs. NF_irrel | 64.1 | [-9.1, 164.9] |
|  |  | conflict vs no conflict | F_rel vs. NF_irrel | 57.2 | [2.9, 109.8] |
|  |  | conflict vs. control | F_rel vs. all | 8.9 | [-52.2, 47.1] |
|  |  | no conflict vs. control | F_rel vs all | 65.7 | [-8.6, 111.5] |
|  |  | conflict vs no conflict | F_rel vs. all | 58 | [-2.6, 99.8] |
|  | 3rd quartile | conflict vs. control | F_rel vs. NF_irrel | 17.6 | [-48.8, 103.7] |
|  |  | no conflict vs. control | F_rel vs. NF_irrel | 80.5 | [7, 174.2] |
|  |  | conflict vs no conflict | F_rel vs. NF_irrel | 61.9 | [5.9, 124.2] |
|  |  | conflict vs. control | F_rel vs. all | 15 | [-29.5, 53] |
|  |  | no conflict vs. control | F_rel vs all | 77.7 | [8.9, 124] |
|  |  | conflict vs no conflict | F_rel vs. all | 61.3 | [3.3, 111.1] |
|  | 4th quartile | conflict vs. control | F_rel vs. NF_irrel | 124.9 | [38.8, 185.4] |
|  |  | no conflict vs. control | F_rel vs. NF_irrel | 144.7 | [65.8, 196.6] |
|  |  | conflict vs no conflict | F_rel vs. NF_irrel | 16.8 | [0.8, 51] |
|  |  | conflict vs. control | F_rel vs. all | 100.5 | [38.4, 122.4] |
|  |  | no conflict vs. control | F_rel vs all | 123.6 | [73.9, 131.7] |
|  |  | conflict vs no conflict | F_rel vs. all | 20.1 | [2.4, 63.8] |
| All interactions executed during all test attempts without the action that led directly to the solution | overall time | conflict vs. control | F_rel vs. NF_irrel | 42.8 | [-44.8, 143.1] |
|  |  | no conflict vs. control | F_rel vs. NF_irrel | 88.8 | [7.2, 185.1] |
|  |  | conflict vs no conflict | F_rel vs. NF_irrel | 48.1 | [-14.8, 121.4] |
|  |  | conflict vs. control | F_rel vs. all | 41.4 | [-13, 90.1] |
|  |  | no conflict vs. control | F_rel vs all | 89.9 | [5.3, 124.1] |
|  |  | conflict vs no conflict | F_rel vs. all | 43.9 | [-27, 98.6] |
|  | 1st half | conflict vs. control | F_rel vs. NF_irrel | -1.4 | [-82.6, 97.1] |
|  |  | no conflict vs. control | F_rel vs. NF_irrel | 58.5 | [-20.3, 160.6] |
|  |  | conflict vs no conflict | F_rel vs. NF_irrel | 60.5 | [-2.3, 130.9] |
|  |  | conflict vs. control | F_rel vs. all | 6.4 | [-51.7, 43.4] |
|  |  | no conflict vs. control | F_rel vs all | 54.5 | [-16.4, 108.9] |
|  |  | conflict vs no conflict | F_rel vs. all | 49.1 | [-11.6, 100.9] |
|  | 2nd half | conflict vs. control | F_rel vs. NF_irrel | 68 | [-8.1, 151.4] |
|  |  | no conflict vs. control | F_rel vs. NF_irrel | 120.2 | [44.7, 194.4] |
|  |  | conflict vs no conflict | F_rel vs. NF_irrel | 53.9 | [7.7, 104.6] |
|  |  | conflict vs. control | F_rel vs. all | 55.1 | [2.5, 97.9] |
|  |  | no conflict vs. control | F_rel vs all | 115.6 | [43.4, 131] |
|  |  | conflict vs no conflict | F_rel vs. all | 55.9 | [3, 104.4] |
|  | 1st quartile | conflict vs. control | F_rel vs. NF_irrel | -1.8 | [-64, 89] |
|  |  | no conflict vs. control | F_rel vs. NF_irrel | 24.5 | [-40.9, 117.2] |
|  |  | conflict vs no conflict | F_rel vs. NF_irrel | 26.5 | [-26.3, 81.6] |
|  |  | conflict vs. control | F_rel vs. all | 9.8 | [-40.6, 42.3] |
|  |  | no conflict vs. control | F_rel vs all | 22.3 | [-32.1, 73.8] |
|  |  | conflict vs no conflict | F_rel vs. all | 13.1 | [-27, 62.5] |
|  | 2nd quartile | conflict vs. control | F_rel vs. NF_irrel | 6 | [-66.7, 102] |
|  |  | no conflict vs. control | F_rel vs. NF_irrel | 61.9 | [-12.8, 163.4] |
|  |  | conflict vs no conflict | F_rel vs. NF_irrel | 55.3 | [0.4, 112.4] |
|  |  | conflict vs. control | F_rel vs. all | 8.2 | [-54.9, 47.4] |
|  |  | no conflict vs. control | F_rel vs all | 60.4 | [-14.1, 110.7] |
|  |  | conflict vs no conflict | F_rel vs. all | 53.6 | [-5.6, 98.9] |
|  | 3rd quartile | conflict vs. control | F_rel vs. NF_irrel | 18 | [-46.7, 103.5] |
|  |  | no conflict vs. control | F_rel vs. NF_irrel | 81.2 | [7.2, 176.7] |
|  |  | conflict vs no conflict | F_rel vs. NF_irrel | 61.9 | [4.9, 125] |
|  |  | conflict vs. control | F_rel vs. all | 13.9 | [-29.1, 51.9] |
|  |  | no conflict vs. control | F_rel vs all | 78.3 | [8.2, 124.8] |
|  |  | conflict vs no conflict | F_rel vs. all | 62.4 | [1.1, 114.7 |
|  | 4th quartile | conflict vs. control | F_rel vs. NF_irrel | 99.8 | [6.8, 171.4] |
|  |  | no conflict vs. control | F_rel vs. NF_irrel | 143.9 | [59.3, 197.4] |
|  |  | conflict vs no conflict | F_rel vs. NF_irrel | 39.3 | [11.7, 88.2] |
|  |  | conflict vs. control | F_rel vs. all | 78.7 | [14.2, 111.3] |
|  |  | no conflict vs. control | F_rel vs all | 123.2 | [65.7, 132.2] |
|  |  | conflict vs no conflict | F_rel vs. all | 40.7 | [12.2, 89.3] |
